# Establishing Normative Values for the Supination Resistance Test: An International Cross‐Sectional Study

**DOI:** 10.1002/jfa2.70137

**Published:** 2026-03-02

**Authors:** Gabriel Moisan, Pier‐Luc Isabelle, Álvaro Gómez Carrión, Dominic Chicoine, Nader Farahpour, Ian Griffiths, Ahmed Dami, Jose Manuel Reguera Medina, Sean McBride

**Affiliations:** ^1^ Department of Human Kinetics Université du Québec à Trois‐Rivières Trois‐Rivières Quebec Canada; ^2^ Groupe de Recherche sur les Affections Neuromusculosquelettiques (GRAN) Université du Québec à Trois‐Rivières Trois‐Rivières Quebec Canada; ^3^ Nursing and Podiatry Department Universidad de Málaga Málaga Spain; ^4^ Department of Sport Biomechanics Faculty of Sport Sciences Bu‐Ali Sina University Hamedan Iran; ^5^ Sports and Exercise Medicine William Harvey Research Institute Queen Mary University of London London UK; ^6^ Department of Anatomy Université du Québec à Trois‐Rivières Trois‐Rivières Quebec Canada; ^7^ Sanipie Clinic Utrera Spain; ^8^ Medical University of South Carolina Charleston South Carolina USA

**Keywords:** ankle, biomechanical phenomena, foot, physical examination, podiatry

## Abstract

**Background:**

The supination resistance test quantifies the force required to supinate the foot and ankle. Although it demonstrates good to excellent intra‐ and inter‐rater reliability and shows potential for predicting foot and ankle biomechanics during walking, assessing the biomechanical effects of foot orthoses, and distinguishing between healthy and pathological conditions, the supination resistance test remains underutilised in both clinical and research settings due to the lack of normative reference values. Thus, the primary objective of this study was to establish international normative values for the supination resistance test based on age and sex. A secondary objective was to compare supination resistance across age groups and between sexes.

**Methods:**

In this international cross‐sectional study, supination resistance was measured in 1198 healthy participants aged 18 years and older from North America, the Middle East, and Europe. Supination resistance was compared across age decades and between sexes using a two‐way analysis of covariance, with body mass included as a covariate. Correlation coefficients and coefficients of determination were calculated to examine the relationships between supination resistance, body mass, and the Foot Posture Index.

**Results:**

Supination resistance was greater in males than in females during the 3rd to 5th and 9th decades of life. In males, it increased from the 3rd to the 5th decade and then progressively declined. In females, it increased from the 3rd to the 6th decade, remained stable through the 7th decade, and then dropped sharply. Body mass accounted for 18.1% of the variance in supination resistance, whereas the Foot Posture Index accounted for only 0.3%.

**Conclusions:**

The international clinical reference values established for the supination resistance test, stratified by age and sex, carry important clinical implications and may support clinicians in screening, monitoring, and managing foot and ankle musculoskeletal conditions. Future research should investigate whether deviations from these normative values represent a risk factor for the development of musculoskeletal injuries and explore the relationship between restoring supination resistance to normative levels and the reduction of clinical symptoms.

**Abbreviations**
FPI: Foot Posture Index
SRT: Supination Resistance Test

## 1 Background

Foot and ankle injuries are common in the general population and are often debilitating [1, 2]. Conditions such as progressive collapsing foot deformity and chronic ankle instability have significant functional consequences, reduce quality of life, and can lead to long‐term complications including foot and ankle deformities, osteoarthritis, and recurrent injuries [3, 4, 5]. Historically, static orthopaedic tests performed in non‐weight‐bearing or quiet standing positions have been used to identify biomechanical deficits to target during treatment and determine the risk of sustaining such musculoskeletal injuries [6, 7]. However, these tests lack validity, are imprecise, and are unable to predict foot and ankle biomechanics during locomotion, offering limited capacity to predict the risk of developing musculoskeletal disorders [8, 9, 10, 11, 12]. In contrast, orthopaedic tests that assess foot and ankle kinetics (e.g., force or stiffness) represent a promising alternative. Recent studies have demonstrated that such tests are more reliable, reproducible, and can predict locomotor biomechanics, both in healthy individuals and those with musculoskeletal impairments [13, 14, 15, 16, 17, 18, 19, 20].

One such test is the supination resistance test (SRT), first described in 1992 (Kirby & Green). The original version involved a manual estimation of the force required to supinate the foot using the examiner's fingers. Greater supination resistance is hypothesised to reflect increased loading of the anatomical structures involved in pronation control and in the generation of supination moments at the subtalar joint during both static and dynamic conditions [21]. The manual version of the SRT demonstrated fair to good intra‐rater and poor to good inter‐rater reliability [22, 23]. Because the manual SRT provides only qualitative force estimations, its clinical and research utility remains limited. Small changes in supination resistance, especially across sessions, cannot be accurately detected. Payne et al. [24] and Noakes and Payne [23] were the first to propose a quantitative version of the test. Their device consisted of a 25‐mm‐wide strip of non‐stretchable woven fabric anchored laterally to the foot at the calcaneocuboid joint, passing under the foot medially near the talonavicular joint, and connecting to a pulley system and force gauge. Their device demonstrated good test‐retest reliability [23, 24]. Griffiths and McEwan [22] later developed a device that more closely mimicked the manual SRT, using a tension/compression load cell to simulate finger pressure. However, its reliability was lower than the device proposed by Payne, Noakes et al. [23, 24]. Moreover, as both systems lacked portability, their application in clinical and research settings was limited. A portable version of the device (Keystone, Interpod, Australia), similar in design to that of Payne and Noakes, was subsequently validated by McBride and Cheng and Moisan et al. [17, 19, 20]. This version of the SRT demonstrated good to excellent intra‐rater and good inter‐rater reliability and was validated in both healthy participants and individuals with musculoskeletal disorders, such as progressive collapsing foot deformity [17, 19, 20].

The quantitative SRT effectively discriminates between individuals with different musculoskeletal conditions. For example, individuals with progressive collapsing foot deformity (previously named posterior tibialis tendon dysfunction) exhibit greater supination resistance, whereas those with chronic ankle instability exhibited less supination resistance. In contrast, individuals with plantar fasciopathy do not significantly differ from healthy controls [25]. Moreover, supination resistance can be effectively modulated using medial or lateral wedge inclinations [25]. Importantly, unlike traditional static orthopaedic tests, the SRT can predict gait biomechanics [18, 19]. Significant correlations between the SRT and midfoot sagittal and frontal plane moments were observed in healthy individuals [18]. Moisan et al. [19] also observed significant positive correlations, in which higher supination resistance was associated with greater ankle eversion, ankle plantarflexion, midfoot dorsiflexion, and midfoot inversion angles during gait in individuals with progressive collapsing foot deformity. Furthermore, healthy participants with higher supination resistance tend to show greater reduction in ankle eversion when wearing custom foot orthoses [26], whereas individuals with progressive collapsing foot deformity and higher supination resistance demonstrate less eversion reduction [19].

The SRT demonstrates good to excellent reliability, can predict gait biomechanics and foot orthoses effects, and differentiates between pathological and healthy states. Despite these strengths, it remains underutilised due to the absence of normative values. In clinical and research contexts, interpreting test results requires comparison against reliable reference data. This allows clinicians and researchers to determine whether an individual's measurement falls within normal limits for the age and sex. Establishing such normative values is essential to fully leverage the SRT in both clinical and research contexts. Thus, the main objective of this study was to establish international normative values for the SRT based on age and sex. The secondary objective was to compare supination resistance across age groups and between sexes. It was hypothesised that, based on data from our previous research projects, supination resistance would be greater in men and would remain stable from the 3rd to the 6th decade of life before beginning to decline in the 7th decade.

## 2 Methods

### 2.1 Participants

To establish normative values, at least 50 participants per group are recommended to reduce variability [27]. Given that this study includes seven age groups and two sexes per age group (a total of 14 subgroups), a minimum of 700 participants was required. However, since the variability of normative data continues to decrease significantly up to 300 participants per subgroup [27], participants across all age groups and sexes were recruited until the required number was met in all subgroups. Thus, 1198 participants were included in this study. Considering the high number of participants required for recruitment, a two‐fold approach was used. First, datasets were acquired from the authors' previous work using the same tool (Keystone Device) and the same method (USA, *n* = 46; Canada, *n* = 108) [17, 18, 20, 25, 28, 29, 30, 31]. Second, 1044 individuals were recruited between January 2024 and October 2025 from North America (Canada, *n* = 403), Europe (Spain, *n* = 343), and the Middle East (Iran, *n* = 298) to participate in this international multicentre study. In Canada, participants were recruited from the outpatient podiatry clinic at the Université du Québec à Trois‐Rivières in Trois‐Rivières. Recruitment efforts were also extended through social media invitations. Additionally, data were collected during two provincial conferences: the Quebec College of Podiatry Conference held in June 2024 and the Integrative Health Conference held in January 2025, both in Saint‐Hyacinthe, Québec, Canada. In Iran, participants were recruited from the Exercise centre specialised in spine care (Mehr Corporation, Hamedan, Iran), the student and staff population of Bu‐Ali Sina University, and local recreational centres. In Spain, participants were recruited from the biomechanics and podiatry unit of the San Agustín Hospital (Dos hermanas, Sevilla, Spain) and from the Sanipie Clinic podiatry centre (Utrera, Seville, Spain).

To be included in the study, all participants needed to be 18 years or older. The exclusion criteria were as follows: experiencing a lower limb musculoskeletal disorder within 3 months of data collection, having a history of a lower limb orthopaedic surgery, being diagnosed with a neuromuscular disorder that could alter balance control or foot and ankle morphology (e.g., multiple sclerosis, Parkinson's disease, stroke), or ongoing pregnancy.

The study protocol was approved by the Université du Québec à Trois‐Rivières Ethic Committee (CER‐23‐305‐07; Canada, Iran) and Bioética y Bioseguridad de Universidad de Extremadura (77/2024, Spain). All participants provided their written consent before the experimentation.

### 2.2 Protocol

Demographic data, consisting of age, sex, body mass, height, and foot posture assessed with the Foot Posture Index (FPI) [32] was recorded (see Table 1).

**TABLE 1:** Demographic data.

Age group (yrs)	Sex	Age (yrs)	Mass (kg)	Height (m)	FPI	Raw supination resistance (*N*)
18–29	Male (*n* = 172)	23.7 (3.9)	82.0 (15.8)	1.79 (0.07)	4.2 (3.7)	97.8 (23.9)
	Female (*n* = 223)	23.0 (2.6)	64.2 (12.2)	1.64 (0.06)	4.2 (3.2)	78.5 (19.0)
30–39	Male (*n* = 74)	34.3 (3.1)	87.9 (17.3)	1.78 (0.07)	4.3 (3.5)	108.0 (27.9)
	Female (*n* = 85)	34.1 (2.8)	67.5 (12.3)	1.64 (0.06)	3.0 (2.8)	85.7 (19.0)
40–49	Male (*n* = 58)	44.9 (2.9)	84.9 (13.2)	1.77 (0.07)	3.9 (3.0)	111.5 (29.7)
	Female (*n* = 102)	43.8 (2.5)	71.0 (13.6)	1.63 (0.06)	3.9 (3.0)	91.2 (20.4)
50–59	Male (*n* = 67)	53.6 (2.5)	82.9 (11.7)	1.74 (0.07)	4.1 (4.0)	105.3 (28.7)
	Female (*n* = 80)	54.4 (3.0)	71.8 (16.7)	1.63 (0.06)	3.3 (3.2)	94.3 (18.1)
60–69	Male (*n* = 57)	64.2 (2.9)	86.4 (17.0)	1.73 (0.06)	4.0 (3.1)	97.6 (23.3)
	Female (*n* = 75)	63.4 (2.6)	71.6 (16.2)	1.60 (0.07)	3.8 (3.0)	94.5 (20.8)
70–79	Male (*n* = 54)	74.9 (2.7)	83.2 (14.0)	1.68 (0.07)	4.7 (3.5)	93.3 (25.2)
	Female (*n* = 51)	74.5 (3.1)	71.1 (13.5)	1.57 (0.06)	5.5 (3.2)	86.7 (22.4)
80+	Male (*n* = 50)	83.7 (4.1)	81.7 (13.4)	1.68 (0.07)	4.3 (3.1)	82.6 (22.3)
	Female (*n* = 50)	84.7 (3.7)	70.7 (12.3)	1.53 (0.08)	5.4 (2.7)	66.8 (14.9)

### 2.3 Supination Resistance Test

**FIGURE 1:**
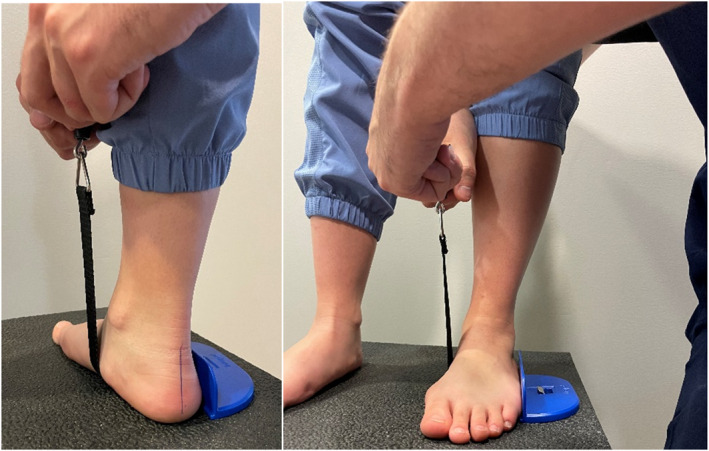
Positioning of the supination resistance test strap, shown from postero‐medial (left) and anterior (right) views.

Supination resistance data were collected using the validated method with the Keystone device (Interpod, Australia) [17, 19, 20] (see Figure 1). Initially, participants were instructed to take five steps in place to assume a relaxed natural stance, to look straight ahead, and to distribute their weight evenly between both feet. A non‐elastic strap, 25 mm in width, was then positioned under the foot, extending from the calcaneocuboid joint to the posteromedial region of the navicular tuberosity. The strap was anchored on the lateral side of the foot, whereas the force gauge from the device was positioned on the medial side. The evaluator applied a steady upward traction on the device at a constant velocity until hindfoot inversion was observed. Once the applied force stabilised, the device locked automatically, and the corresponding force value was recorded. Participants were instructed not to assist or oppose the traction force during data collection. Three supination resistance measures were taken for each foot. Data were measured in kilogrammes and converted into Newtons (N). Before collecting data, coordination meetings were undertaken to ensure the consistency of the experimental protocol across countries and raters. All raters completed at least 200 supination resistance measurements prior to data collection to familiarise themselves with the protocol and ensure the acquisition of reliable data.

### 2.4 Statistical Analysis

Data were analysed with SPSS‐29.0.2.0 (IBM Corporation, Armonk, NY, USA). The mean supination resistance value from three trials was calculated for each participant. Data were averaged according to age groups: 18 to 29, 30 to 39, 40 to 49, 50 to 59, 60 to 69, 70 to 79, and 80+ years. Supination resistance data were also disaggregated according to sex for a total of 14 subgroups. The normality of the distribution for demographic and supination resistance data was evaluated using visual methods, including histograms and probability graphs.

Given the normal distribution of the supination resistance data, pooled supination resistance data across age groups and sexes were compared between the right and left foot using a paired *t*‐test. Considering the large sample size, the level of statistical significance was set at *α* = 0.050 and *d* ≥ 0.50 for this analysis. As no statistically significant difference was found between feet, only the dominant foot (foot used to kick a ball) was included in subsequent analyses.

Bivariate regression analyses between supination resistance and body mass were conducted using Pearson's correlation coefficient (*r*) with 95% confidence intervals to identify potential covariates. Bivariate analyses between supination resistance and the FPI score were conducted using Spearman's rank correlation coefficient (*ρ*), as FPI data were not normally distributed. The coefficient of determination (*R* ^2^) was also calculated. Considering the large sample size, the level of statistical significance was set at *α* = 0.050 and r/*ρ* ≥ 0.40 [33]. Correlation coefficients (*ρ*) less than 0.10 were considered negligible; values between 0.10 and 0.39 were considered weak, 0.40 to 0.69 moderate, 0.70 to 0.89 strong, and 0.90 to 1.00 very strong [33].

Considering that body mass was identified as a covariate, a two‐way ANCOVA was performed with “age group” (seven levels) and “sex” (two levels) as independent factors, and body mass as the covariate for the SRT. Estimated marginal means were compared using Bonferroni‐adjusted pairwise comparisons for the main effects of age group and sex, as well as for their interaction. The significance level was set at *α* = 0.050 for these analyses. To facilitate clinical interpretation, supination resistance raw data were also categorised into percentiles stratified by age group and sex: very low (< 2.3), low (2.3–15.9), slightly low (15.9–25.0), normal (25.0–75.0), slightly high (75.0–84.1), high (84.1–97.7), and very high (> 97.7). These percentile cut‐offs were selected to reflect standard statistical boundaries (e.g., ± 2 SD for the extremes and quartile‐based thresholds for intermediate categories), allowing clinically meaningful stratification across subgroups.

## 3 Results

**FIGURE 2:**
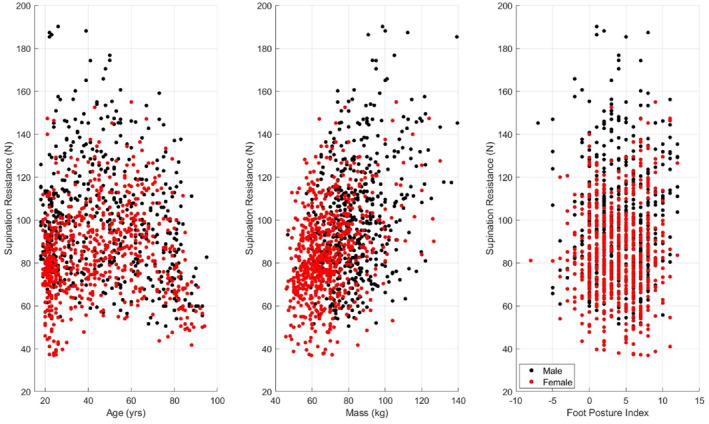
Scatterplots of supination resistance in relation to age (left), mass (centre), and Foot Posture Index (right).

**FIGURE 3:**
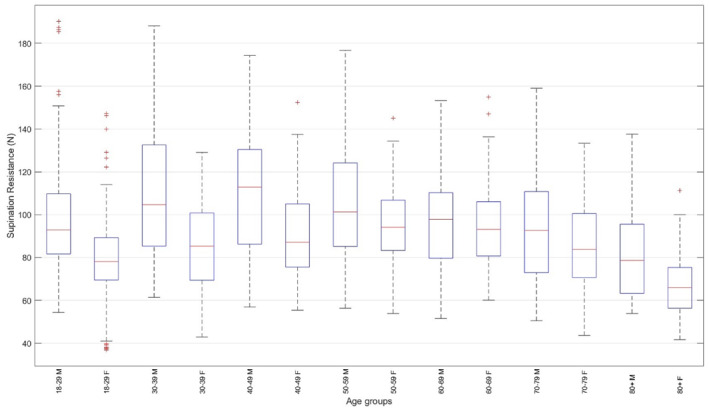
Boxplots of supination resistance across age groups and sexes. Each boxplot illustrates the median, interquartile range, and potential outliers for supination resistance within each subgroup.

For the retrospective data (*n* = 154), supination resistance was only available for the dominant foot. Thus, the between‐feet comparison only included prospectively collected data (*n* = 1044). There was no significant difference in supination resistance between the left and right foot (91.0 ± 24.9 N vs. 92.4 ± 24.1 N, *p* < 0.001, *d* = 0.103). The distribution of the supination resistance data according to age, body mass, and the FPI is displayed in Figure 2, and across age groups and sex in Figure 3.

There was a significant correlation between body mass and supination resistance (*r* = 0.425 (95% CI: 0.377–0.470), *p* < 0.001, *R* ^2^ = 0.181) (see Figure 2). In contrast, the correlation between supination resistance and the Foot Posture Index score was not significant (*ρ* = 0.052, (95% CI: −0.006–0.110), *p* < 0.001, *R* ^2^ = 0.003) (see Figure 2).

### 3.1 Age Group Effects

Estimated marginal means, adjusted for an average body mass of 75.2 kg. A significant age group effect was observed (*F* = 19.9, *p* < 0.001, *η*2 = 0.092). Regardless of sex, supination resistance in the 18–29 age group was lower than in the 30–39 (−6.3 N, *p* = 0.037), 40–49 (−10.7 N, *p* < 0.001) and 50–59 (−9.5 N, *p* < 0.001) age groups, and was higher than in the 80+ age group (15.0 N, *p* < 0.001). Supination resistance in the 30–39 age group was higher than in the 80+ age group (21.3 N, *p* < 0.001). Supination resistance in the 40–49 age group was greater than in the 70–79 (10.9 N, *p* = 0.001) and 80+ (25.7 N, *p* < 0.001) age groups. Supination resistance in the 50–59 age group was greater in the 70–79 (9.6 N, *p* = 0.008) and 80+ (24.5 N, *p* < 0.001) age groups. Supination resistance in the 60–69 (19.9 N, *p* < 0.001) and the 70–79 (14.9 N, *p* < 0.001) age groups was greater than in the 80+ age group.

### 3.2 Sex Effects

There was a significant sex effect (*F* = 19.9, *p* < 0.001, *η*2 = 0.092). Regardless of the age group, male participants exhibited greater supination resistance than female participants (94.9 vs. 88.2 N, *p* < 0.001).

### 3.3 Interaction Effects

There was a significant interaction effect (*F* = 20.1, *p* < 0.001, *η*2 = 0.017). See Figure 3 for boxplots of supination resistance across age groups and sexes.

For male participants, supination resistance in the 18–29 age group was smaller than in the 40–49 age group (−12.1 N, *p* = 0.004) and higher than in the 80+ age group (15.1 N, *p* < 0.001). Supination resistance in the 30–39 age group was higher than in the 70–79 (12.3 N, *p* = 0.027) and 80+ (22.2 N, *p* < 0.001) age groups. Supination resistance in the 40–49 age group was higher than in the 60–69 (14.8 N, *p* = 0.004), 70–79 (17.3 N, *p* < 0.001), and 80+ (27.2 N, *p* < 0.001) age groups. Supination resistance in the 50–59 age group was higher than in the 70–79 (12.1 N, *p* = 0.039) and 80+ (22.0 N, *p* < 0.001) age groups.

For female participants, supination resistance in the 18–29 age group was smaller than in the 40–49 (−9.3 N, *p* = 0.006), 50–59 (−12.0 N, *p* < 0.001), and 60–69 (−12.3 N, *p* < 0.001) age groups. Supination resistance in the 80+ age group was smaller than in the 20–29 (−14.9 N, *p* < 0.001), 30–39 (−20.5 N, *p* < 0.001), 40–49 (−24.2 N, *p* < 0.001), 50–59 (−26.9 N, *p* < 0.001), 60–69 (−27.2 N, *p* < 0.001), and 70–79 (−19.8 N, *p* < 0.001) age groups.

For the same age group, male participants exhibited greater supination resistance than female participants in the 18–29 (10.4 N, *p* < 0.001), 30–39 (11.9 N, *p* < 0.001), 40–49 (13.2 N, *p* < 0.001), and 80+ (10.2 N, *p* = 0.017) age groups.

**FIGURE 4:**
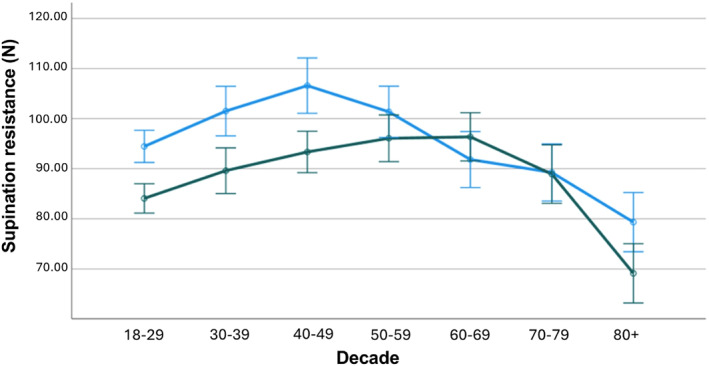
Estimated marginal means for supination resistance. Blue curve: male participants, green curve: female participants. Covariate (Mass) = 75.2 kg.

See Figure 4 for the age and sex group comparisons in supination resistance with the estimated marginal means. See Table 2 for the clinical categories for the supination resistance test according to age and sex.

**TABLE 2:** Clinical categories for the supination resistance test according to age and sex. A one‐pager clinical sheet is available in Supporting Information S1.

Clinical categories for the supination resistance test (*N*)								
Age group (yrs)	Sex	Very low	Low	Slightly low	Normal	Slightly high	High	Very high
18–29	Male (*n* = 172)	< 61.3	61.3–78.5	78.5–81.7	81.7–109.8	109.8–114.1	114.1–185.4	> 185.4
	Female (*n* = 223)	< 38.1	38.1–60.8	60.8–69.4	69.4–89.4	89.4–93.9	93.9–125.9	> 125.9
30–39	Male (*n* = 74)	< 63.0	63.0–77.5	77.5–84.8	84.8–133.1	133.1–138.6	138.6–171.4	> 171.4
	Female (*n* = 85)	< 47.7	47.7–66.8	66.8–69.3	69.3–100.8	100.8–102.7	102.7–128.2	> 128.2
40–49	Male (*n* = 58)	< 57.6	57.6–78.6	78.6–86.2	86.2–130.8	130.8–144.0	144.0–172.9	> 172.9
	Female (*n* = 102)	< 57.4	57.4–72.7	72.7–75.5	75.5–105.0	105.0–114.2	114.2–137.0	> 137.0
50–59	Male (*n* = 67)	< 57.2	57.2–77.3	77.3–84.5	84.5–124.5	124.5–140.3	140.3–175.4	> 175.4
	Female (*n* = 80)	< 54.8	54.8–76.8	76.8–83.2	83.2–106.8	106.8–112.9	112.9–135.8	> 135.8
60–69	Male (*n* = 57)	< 52.5	52.5–76.0	76.0–79.4	79.4–110.8	110.8–119.1	119.1–150.9	> 150.9
	Female (*n* = 75)	< 60.5	60.5–71.8	71.8–80.7	80.7–107.1	107.1–115.5	115.5–149.1	> 149.1
70–79	Male (*n* = 54)	< 50.9	50.9–67.4	67.4–72.5	72.5–111.1	111.1–118.3	118.3–155.8	> 155.8
	Female (*n* = 51)	< 44.0	44.0–66.1	66.1–70.3	70.3–100.5	100.5–114.5	114.5–132.4	> 132.4
80+	Male (*n* = 50)	< 54.2	54.2–59.8	59.8–63.1	63.1–96.2	96.2–110.6	110.6–137.4	> 137.4
	Female (*n* = 50)	< 42.9	42.9–50.6	50.6–55.9	55.9–75.8	75.8–81.2	81.2–109.4	> 109.4

## 4 Discussion

The main objective of this study was to establish international normative values for the SRT based on age and sex. The secondary objective was to compare supination resistance across age groups and between sexes.

Overall, supination resistance followed a nonlinear sex‐specific trajectory across the lifespan. In males, values increased from the 3rd to the 5th decade, reflecting the period of maximal musculoskeletal strength and stiffness, then declined progressively until the 9th decade. In females, supination resistance increased more gradually, reaching a plateau in the 6th and 7th decades before decreasing sharply thereafter. This trajectory could reflect the combined effects of hormonal, structural, and neuromuscular changes occurring with ageing. The decline observed in both sexes after midlife is consistent with reductions in muscle mass, tendon stiffness, and collagen cross‐linking previously reported in ageing biomechanics literature. The peak observed in females during the 6th decade may also coincide with menopause‐related decreases in oestrogen levels, known to affect collagen synthesis and tendon mechanical properties (see Figures 3, 4). Males exhibited greater supination resistance than females in the 3rd, 4th, 5th, and 9th decades, suggesting a sex‐based difference in mechanical stiffness and load‐bearing capacity across most of adulthood. The absence of significant differences between sexes during the 6th to 8th decades may indicate a convergence in musculoskeletal characteristics during midlife, potentially linked to hormonal and physiological adaptations in both sexes.

The difference between the lower and upper bounds (25th to 75th percentile) of normal supination resistance ranged from 20 to 32 N in females and 28–48 N in males. This highlights that normal supination resistance is not a single fixed value for each decade and sex but rather falls within a narrow range. Clinicians and researchers need to appreciate this range of normal values when using the SRT.

Previous studies have shown that body mass can explain between 12% and 50% of the variance of supination resistance in healthy individuals [20, 22, 23] whereas the Foot Posture Index only explains from less than 0.1%, up to 12% of the variance [22, 23]. In our study, body mass explained 18.1% of the variance of supination resistance, whereas the Foot Posture Index score only explained 0.3%. The discrepancy with previous findings may be explained by the use of the Keystone device, which differs from the equipment used by Griffiths, McEwan [22], and Noakes and Payne [23] to measure supination resistance. However, Moisan et al. [20] used the same protocol and reported that body mass explained 50% of the variance in supination resistance, which is significantly higher than in our study. Their sample size, however, was small compared with the present study and included only participants with low supination resistance (average of 53–62 N). Notably, all of their data were included in our study's sample, which suggests that if they had recruited more participants, the explained variance would have been smaller. Although body mass is a statistically significant factor, the majority (81.9%) of the variance in supination resistance remains unexplained by body mass alone, suggesting that other intrinsic and extrinsic variables contribute meaningfully to supination resistance. Given this, we chose to report normative data based on the raw supination resistance values rather than those normalised to body weight as previously used [25, 26, 30]. Nonetheless, normalised supination resistance values and percentiles are provided in the Supporting Information S1 for reference. Considering the moderate correlation between body mass and supination resistance found in our study, we suggest caution when performing the SRT in individuals with a very low or a very high body mass compared with the reported means in this study. Using the mass‐normalised values of supination resistance (see Supporting Information S1) could be better suited in these populations. Consistent with prior research, our findings reaffirm that foot morphology, as assessed by the FPI, is not associated with supination resistance. In practical terms, foot type assessed with the FPI, whether planus or cavus, does not predict supination resistance and should not be used as a surrogate measure. In clinical contexts, these international SRT norms may be used to anticipate the biomechanical effects of foot orthoses and to guide the selection of orthotic features tailored to individual needs [19, 26]. They may also serve to monitor changes in supination resistance before and after an intervention in individuals with foot and ankle musculoskeletal disorders, with the aim of restoring supination resistance toward values consistent with these normative data.

### 4.1 Research Perspectives

Although not assessed in the present study, previous research indicates that the transverse planar position of the subtalar joint axis may be a stronger predictor of supination resistance, with a coefficient of determination of *R* ^2^ = 0.35 [24]. Future studies should aim to further explore this relationship using newer digital methods of estimating the subtalar joint axis location [34]. Future research should also determine whether a deviation in supination resistance from the established normative values constitutes a risk factor for the development of musculoskeletal injuries, such as lateral ankle sprain or progressive collapsing foot deformity. For example, Moisan et al. [25] showed in their cross‐sectional case‐control study that individuals with progressive collapsing foot deformity exhibit greater supination resistance. However, the cross‐sectional design of that study precludes causal inference. Prospective longitudinal investigations are therefore needed to clarify whether elevated supination resistance predisposes individuals to, or results from, such musculoskeletal disorders. It would also be pertinent to evaluate the effectiveness of conservative interventions, such as foot orthoses and strengthening exercises, as well as surgical treatments, in normalising supination resistance in individuals with foot and ankle musculoskeletal disorders. Furthermore, research should examine the relationship between the normalisation of supination resistance and the reduction of clinical symptoms, particularly pain, during these treatments. Finally, individuals with progressive collapsing foot deformity who have lower supination resistance demonstrate greater biomechanical effects to their foot and ankle when wearing foot orthoses [19]. It would be important to study whether supination resistance can predict the clinical effects of foot orthoses, such as pain reduction and improvement in function. This relationship would help determine whether supination resistance could serve as a predictor of therapeutic success in orthotic therapy.

### 4.2 Limitations

The main limitation of this study is the uneven distribution of the population sample across countries. Ideally, an equal number of men and women across all age groups would have been recruited in each country. However, due to the complexity of recruitment and the limited access to potential participants in certain countries, this was not feasible. Potential cultural and/or lifestyle differences might have influenced foot morphology, function and biomechanics across countries. A further limitation is that all data were not collected by the same rater due to logistical and financial challenges. Nevertheless, given the good inter‐rater reliability of the SRT [20], we are confident that our normative values remain valid despite these limitations. Another limitation of these normative data is specific to the Keystone device. Therefore, if clinicians or researchers use other tools or equipment to record supination resistance and want to use these normative values, they should first evaluate the concurrent validity of their measurements in comparison to the Keystone device.

## 5 Conclusions

At equivalent body mass, males demonstrate greater supination resistance than females during the 3rd, 4th, 5th, and 9th decades of life. In males, supination resistance increases from the 3rd to the 5th decade, followed by a gradual decline with advancing age. In females, it rises from the 3rd to the 6th decade, remains relatively stable during the 7th decade, and then decreases sharply after the age of 70. The normative reference values established in this study provide, for the first time, an international benchmark for interpreting supination resistance measurements. They have important clinical implications, offering clinicians an evidence‐based framework to screen, monitor, and manage foot and ankle musculoskeletal conditions across diverse populations and age groups. By comparing individual measurements to these norms, clinicians may also identify atypical supination resistance early, potentially allowing for the prevention of injuries or the implementation of targeted interventions before musculoskeletal disorders develop.

## Author Contributions

**Gabriel Moisan:** conceptualization, formal analysis, funding acquisition, investigation, methodology, project administration, supervision, visualization, writing – original draft, writing – review and editing. **Pier‐Luc Isabelle:** funding acquisition, investigation, writing – review and editing. **Álvaro Gómez Carrión:** funding acquisition, investigation, writing – review and editing. **Dominic Chicoine:** funding acquisition, investigation, writing – review and editing. **Nader Farahpour:** funding acquisition, investigation, writing – review and editing. **Ian Griffiths:** funding acquisition, writing – review and editing. **Ahmed Dami:** investigation, writing – review and editing. **Jose Manuel Reguera Medina:** investigation, writing – review and editing. **Sean McBride:** methodology, funding acquisition, writing – review and editing.

## Funding

This study was funded by the Ordre des Podiatres du Québec, the Fondation de l’UQTR, the Syndicat des chargés de cours de l’UQTR, and the Fonds de Recherche du Québec‐Santé (#347697).

## Ethics Statement

The study protocol was approved by the Université du Québec à Trois‐Rivières Ethic Committee (CER‐23‐305‐07; Canada, Iran), and Bioética y Bioseguridad de Universidad de Extremadura (77/2024, Spain). All participants provided their written consent before the experimentation.

## Consent

The authors have nothing to report.

## Conflicts of Interest

The authors declare no conflicts of interest.

## Supporting information


Supporting Information S1

Supporting Information S2

## Data Availability

The datasets used and/or analysed during the current study are available from the corresponding author on reasonable request.
