# Supplementary material for: Establishing Normative Values for the Supination Resistance Test: An International Cross‐Sectional Study
Source: J Foot Ankle Res. 2026 Mar 2;19(1):e70137. doi: 10.1002/jfa2.70137 (PMC12953718; doi:10.1002/jfa2.70137)
Supplement: Supplementary file 1 — Supporting Information S1 [file JFA2-19-e70137-s001.pdf]

International Normative Reference Values for the Supination Resistance Test (SRT)

The Supination Resistance Test (SRT) quantifies the force (in newtons) required to supinate the foot and ankle. These reference values were established from 1198 healthy adults (North America, Europe, Middle East), stratified by age and sex. Use this chart to interpret SRT results in clinical practice.

Age (yrs)	Sex	Very low	Low	Slightly low	Normal	Slightly high	High	Very high
18-29	Male	<61.3	61.3-78.5	78.5-81.7	81.7-109.8	109.8-114.1	114.1-185.4	>185.4
	Female	<38.1	38.1-60.8	60.8-69.4	69.4-89.4	89.4-93.9	93.9-125.9	>125.9
30-39	Male	<63.0	63.0-77.5	77.5-84.8	84.8-133.1	133.1-138.6	138.6-171.4	>171.4
	Female	<47.7	47.7-66.8	66.8-69.3	69.3-100.8	100.8-102.7	102.7-128.2	>128.2
40-49	Male	<57.6	57.6-78.6	78.6-86.2	86.2-130.8	130.8-144.0	144.0-172.9	>172.9
	Female	<57.4	57.4-72.7	72.7-75.5	75.5-105.0	105.0-114.2	114.2-137.0	>137.0
50-59	Male	<57.2	57.2-77.3	77.3-84.5	84.5-124.5	124.5-140.3	140.3-175.4	>175.4
	Female	<54.8	54.8-76.8	76.8-83.2	83.2-106.8	106.8-112.9	112.9-135.8	>135.8
60-69	Male	<52.5	52.5-76.0	76.0-79.4	79.4-110.8	110.8-119.1	119.1-150.9	>150.9
	Female	<60.5	60.5-71.8	71.8-80.7	80.7-107.1	107.1-115.5	115.5-149.1	>149.1
70-79	Male	<50.9	50.9-67.4	67.4-72.5	72.5-111.1	111.1-118.3	118.3-155.8	>155.8
	Female	<44.0	44.0-66.1	66.1-70.3	70.3-100.5	100.5-114.5	114.5-132.4	>132.4
80+	Male	<54.2	54.2-59.8	59.8-63.1	63.1-96.2	96.2-110.6	110.6-137.4	>137.4
	Female	<42.9	42.9-50.6	50.6-55.9	55.9-75.8	75.8-81.2	81.2-109.4	>109.4

UQTR Université du Québec à Trois-Rivières

UNIVERSIDAD DE MÁLAGA

دانشگاه بوعلی سینا

Queen Mary University of London

MUSC MEDICAL UNIVERSITY of SOUTH CAROLINA
